# Swasthya Pahal (Health for All) Using a Sustainable, Multisector, Accessible, Affordable, Reimbursable, and Tailored Informatics Framework in Rural and Urban Areas of Chennai, Tamil Nadu: Protocol for a Quantitative Study

**DOI:** 10.2196/39950

**Published:** 2025-02-06

**Authors:** Ashish Joshi, Krishna Mohan Surapaneni, Ashoo Grover, Harpreet Kaur, Sofia Rani Saggu, Doilyn Oliveira

**Affiliations:** 1 School of Public Health University of Memphis Memphis, TN United States; 2 Panimalar Medical College Hospital & Research Institute Chennai India; 3 Indian Council of Medical Research New Delhi India; 4 Foundation of Healthcare Technologies Society New Delhi India

**Keywords:** interventions, Swasthya Pahal, acceptance, health behavior, NCDs risks, self-management, healthcare, noncommunicable disease

## Abstract

**Background:**

Noncommunicable diseases (NCDs) require a longer period of care, for which health care systems must acquire technologically advanced solutions to enhance patient care. Swasthya Pahal (health for all) is an innovative, interactive, multilingual, stand-alone, internet-enabled computer-based program that aims to improve the self-management of NCDs.

**Objective:**

This study aims to enhance the self-management of chronic NCDs (diabetes, hypertension, high cholesterol, and obesity) by determining the usefulness, acceptance, and effectiveness of the Swasthya Pahal program in hospital and community settings in both rural and urban areas of Chennai, Tamil Nadu. This objective can be met by generating risk factor profiles of individuals enrolled and enhancing their self-management of NCDs using a portable health information kiosk that uses the Sustainable, Multisector, Accessible, Affordable, Reimbursable, and Tailored (SMAART) model.

**Methods:**

A quantitative study will be conducted on a convenient sample of 2800 individuals from selected hospital and community settings in rural (n=1400) and urban areas (n=1400) in Chennai, Tamil Nadu. Data will be collected on sociodemographics, health behaviors, and clinical status, as well as knowledge, attitudes, and practices. Objective assessments such as weight, blood pressure, and random blood sugar levels will be measured. In addition, the usefulness, acceptance, and effectiveness of the Swasthya Pahal program will be determined.

**Results:**

Results will be summarized using descriptive analysis. Appropriate bivariate and multivariate regression analysis will be performed to determine the predictors of the outcome variables of usefulness, acceptance, and effectiveness of Swasthya Pahal in wider settings. All analyses will be performed using SAS (version 9.1; SAS Institute), and the results will be reported as 95% CI values and *P*<.05.

**Conclusions:**

The study proposes to enhance the self-management of NCDs in both rural and urban community settings through the implementation of the Swasthya Pahal program based on the SMAART informatics framework. The study aims to understand the implementation, acceptability, and usability of Swasthya Pahal among a diverse sample of people in urban and rural settings.

**International Registered Report Identifier (IRRID):**

PRR1-10.2196/39950

## Introduction

### Background

Noncommunicable diseases (NCDs), which include heart disease, stroke, cancer, diabetes, and chronic lung disease, are the leading causes of illness worldwide. This has been identified as one of the most significant challenges to sustainable development in the 21st century [1]. According to the World Health Organization, NCDs account for approximately 38 million (68%) of all deaths worldwide and approximately 5.87 million (60%) of all deaths in India [2]. The Sustainable Development Goal target 3.4 is to reduce premature mortality from NCDs by one-third by 2030 relative to 2015, as well as to promote mental health and well-being [3]. India faces a significant burden of NCDs, which poses a challenge in achieving Sustainable Development Goal 3 Good Health and Well-Being. In recent years, India has experienced demographic and epidemiological shifts, resulting in a shift from infectious diseases to NCDs. The World Health Organization reports that 1 in every 4 Indians is at risk of dying from an NCD before the age of 70 years [4]. If timely NCD prevention and control measures are not implemented, the total annual number of NCD deaths will rise to 55 million by 2030 [5].

In the current era, information and communication technologies (ICTs) serve as health systems, promoting, restoring, or maintaining health. They should be used for the prevention and control of both communicable and NCDs at multiple levels and in a variety of ways [4]. The use of ICT in a health-promoting lifestyle behavior program improves health behaviors that are crucial in the prevention of both communicable and noncommunicable diseases. eHealth is defined as the application of ICT in the world of health care. It includes mobile health, telemedicine, health information systems, electronic health records, and many more [6]. These technologies can be presented and accessed in a variety of ways, including web-based applications, mobile phone and alert systems, and phone and videoconferencing. They have been identified as effective measures for improving patient skills and knowledge, increasing the likelihood of healthy behavior [7]. It can provide both low-cost and high-quality services. Health information products can be designed to suit the needs of the community by incorporating high levels of interaction and accessibility. Technological solutions play an important role in health care systems by assisting individuals in self-checking their health status and providing low-cost, accessible, and affordable solutions to meet the demands of local communities across geographical locations, particularly those in resource-poor settings [8]. Studies have shown that the use of ICT through mobile health in India has benefitted both urban and rural healthy and unhealthy (“disease”) population groups. [9-11] Health kiosks will continue to play an important role in the digital health scene for the foreseeable future. One of the advantages of telehealth kiosks is that they bring medical and expert care to remote locations that medical practitioners rarely visit. In nations such as India, these locations could be distant rural areas with inadequate infrastructure [12]. A pilot study of the Swasthya Pahal (health for all) program, which is an advanced community-based program facilitated by an interactive, multilingual, standalone, and internet-enabled program, has highlighted the improvement of self-management of NCDs among police personnel [13]. The program was implemented through a digital, interactive touch screen platform Sustainable, Multisector, Accessible, Affordable, Reimbursable, and Tailored (SMAART) model. The potential outcome of the SMAART platform is that it is a personalized self-management action plan that enables easier self-management and that is backed up by continuous monitoring, interactive health information, and collaborative decision-making [8]. The pilot study had significant findings: the applicability of the study and its use beyond a purposive population group needed to be further studied. The program is based on the Population Health Informatics framework; to the best of our knowledge, this framework is now used to address the burden of NCDs in both urban and rural economic groups. This study aims to evaluate the usability of a portable health information kiosk that uses the SMAART framework to address the burden of NCDs by determining the usefulness, acceptance, and effectiveness of Swasthya Pahal in hospital and community settings of both rural and urban areas of Chennai, Tamil Nadu.

### Objectives

The objectives of the research are as follows:

1. To use the digital health intervention Swasthya Pahal to assess the risk factor profile of NCDs in individuals from hospital and community settings in rural and urban areas of Chennai, Tamil Nadu.
2. To assess individuals’ Knowledge, Attitudes, and Practices (KAP) of their health status and self-management of chronic NCDs (diabetes, hypertension, high cholesterol, and obesity) through the Swasthya Pahal program among individuals from hospitals and community settings in rural and urban areas of Chennai, Tamil Nadu.
3. To investigate the usefulness and acceptance of the Swasthya Pahal program among individuals from the hospital and community settings in rural and urban areas of Chennai to enhance self-management of NCDs.

## Methods

### Study Design

The quantitative pre-post study will recruit 2800 study participants from the community and hospital settings in urban and rural areas of Chennai, Tamil Nadu. Individuals will have access to a touch screen device to register their health data in their respective languages. Subjective data on sociodemographic details, health behaviors, clinical status, and KAP will be gathered. Objective data will include measurements of height, weight, blood pressure, and blood glucose levels. Individuals will be randomly assigned to the intervention and control group. A total of 20% of the rural and urban samples will be assigned to the control group (total N=560; n=280 from each urban and rural area) and intervention group (total N=560; n=280 from each urban and rural area). Those in the intervention group will have access to tailored messages through the use of multiple interactive digital formats using podcasts and SMS. Those in the control group will have access to standard of care and will be provided with a booklet in English and in the local dialect to self-manage the burden of NCDs. Individuals will receive interactive messages for 3 months on a daily or weekly basis. The message content and delivery will be tailored (based on sociodemographics, KAP, user preferences, and disease conditions) to meet the specific needs of the individuals. Monthly assessments will be done at month(s) 1, 2, and 3. At 6 months, retention analysis will be done for Knowledge. In addition, the usefulness, acceptance, and effectiveness of Swasthya Pahal will be assessed postintervention.

### Study Setting and Study Population

The study population will be from hospital and community settings of selected rural and urban areas of Chennai, Tamil Nadu, namely Kannur, Pannur, Thirumanikuppam, Thodukadu, Kottaiyur, Nemili, Mannur, Kiloy, Ulundai, Narasamangalam, Karanai, Panimalar Medical College Hospital and the Research Institute and Rural Health Training Centre Chennai, and Maligaipattu village.

### Sampling and Recruitment

Participants will be recruited using the nonprobability convenience sampling method from selected urban and rural sites of Chennai, Tamil Nadu. Data will be gathered from 2800 participants (1400 each from urban and rural sites).

Inclusion criteria were participants above the age of 18 years and those consenting to participate and exclusion criteria were individuals below the age of 18 years and those who would not give their consent.

### Data Collection—Structured and Semistructured Questionnaire

#### Overview

A structured questionnaire will be used to determine the risk factor profile of NCDs in individuals from selected rural and urban areas of Chennai, Tamil Nadu. For objectives 1 and 2, data will be collected on the portable health information kiosk. Individuals will be enrolled in the Swasthya Pahal program with a unique code that will generate a SMAART health card. Information will be recorded electronically, that is, subjective data that captures sociodemographic profile, clinical status, information on health behavior, KAP of individuals, and objective data such as BMI, blood pressure, and blood glucose levels will be measured. After 3 months of intervention, a posttest will be conducted on KAP, and then a crossover of intervention mode is done. For objective 3, after the dual intervention, KAP will be conducted and the acceptance and usability of the program will be assessed by 2 tools: the Client Satisfaction Questionnaire and System Usability Scale (annexures 2 and 3 in Multimedia Appendix 1). The tool will be translated into the local language (Tamil). A small group of respondents will pilot test the study tool in the local language to ensure the clarity of the questions asked.

#### Variables Assessed

The following variables will be assessed in the study: variables A1, A2, A3, and A4 will be assessed with respect to objectives 1 and 2, and variables B1 and B2 will be assessed with respect to objective 3.

#### A1—Sociodemographic Details

Data will be gathered on the study participant’s age, gender, region of residence, and education level.

#### A2—Health Behavior

Data on history and current smoking and alcohol consumption will be compiled. Data on the family history of consumption will also be collected.

#### A3—Clinical Status Details

This will include information on anthropometric measurements such as height (as measured by a Stadiometer), weight (as measured by a standard technique), and BMI. A sphygmomanometer will be used to take blood pressure readings. A glucometer will be used to measure blood glucose levels.

#### A4—Knowledge, Attitudes, and Practices

These data will collect information on an individual’s understanding of their BMI, blood pressure, and blood sugar level. The information gathered will determine whether the individual is currently receiving treatment for any disease risks and what treatments have already been followed (eg, diet, medicine, and physical activity). The tool also captures the individual’s level of understanding of obesity, hypertension, and diabetic risk factors. It also acquires information on individuals receiving treatment for any of the illness risks and information on individuals’ understanding of their family’s health history.

#### B1—Tool for Acceptance: Client Satisfaction Questionnaire

This questionnaire includes a total of 8 questions on a 4-point Likert scale that contains questions related to the accessibility of the computer-based software Swasthya Pahal.

#### B2—Tool for Usability: System Usability Scale

The System Usability Scale consists of 10 questions on which individuals rate the system on a 5-point Likert scale based on their opinion of the system’s usefulness.

#### Data Collection, Data Entry, and Quality Assurance

To ensure efficient and high-quality data collection and processing, the following data management protocol will be followed: a well-defined study manual will be prepared, and a well-trained data collection team will be in charge of data collection, data entry, and data security procedures. Each question will be explained clearly by the data collector, and any doubt or confusion regarding the question will be addressed immediately. The data will be electronically recorded using the Swasthya Pahal (Health for All) software.

#### Data Security and Privacy

Regular backups will ensure data security by storing data files in one password-protected computer or laptop and deleted from other systems. The data files will have a unique password that only the concerned research team will be able to access. Data will be kept for 15 years after the study is completed in case it is needed to validate research findings, set priorities, or be re-analyzed by another researcher [14].

### Data Analysis

The collected information will be entered in SAS (version 9.1; SAS Institute) and the results were reported as 95% CI values and *P*<.05. The data will be summarized by using frequency, percentage, mean, and SD values. Appropriate bivariate and multivariate analyses will be done. A *t* test to compare means between the continuous variables and a categorical dependent variable will be conducted. A chi-square analysis will be performed to determine whether the two independent variables are related.

### Project Timeline and Milestones

A detailed research plan and schedule timeline of the tasks involved in the study are presented in Table 1.

**Table 1:** Scheduled timeline of the tasks in the Swasthya Pahal.

Task	Months									
	1	2	3	4	5-7	8-10	11-13	13-15	16-19	20-24
Review of the literature, initial design, and planning of the study	✓	✓								
Development of study protocol and ethical approval		✓								
Approval of the study		✓								
Development of the study tool		✓								
Review and revision of the study tool by the research team			✓							
Training of the data collection team			✓							
Pilot testing of Swasthya Pahal computer-based tool			✓							
Initial data analysis, results, discussion, and dissemination of the pilot study				✓						
Deployment of the Swasthya Pahal intervention and baseline data collection					✓					
Recruitment of the target sample and assignment into intervention and control groups					✓					
Post assessment at 1, 2, 3, and 6 months					✓	✓	✓			
Reviewing collected data by the research team								✓	✓	
Data analysis									✓	
Results and Discussion and Conclusion										✓
Dissemination										✓

### Informed Consent

The research team will provide the informed consent form approved by the Institutional Review Board (IRB) to all eligible participants in the study (Multimedia Appendix 1). The team will explain the study details, the time commitment, and the benefits of the study results to the participants. Those who are willing to participate and provide consent will be enrolled in the study. If any participant is uneducated, ethical consent will be audio recorded. Furthermore, uneducated participants will be instructed on how to complete the questionnaire in the local Indian dialect, increasing the study’s usefulness and generalizability. Data privacy and patient confidentiality will be assured. The study participants will have the right to withdraw from the study at any time.

### Ethical Considerations

The study with protocol number PMCHRI-IHEC-058 gained approval from the Panimalar Medical College Hospital and Research Institute Institutional Human Ethics Committee (Central Drugs Standard Control Organization Registration number ECR/1399/Inst/TN/2020) in February 2022 with approval number PMCH&RI/IHEC/20221/021 dated February 18, 2022. The study will be conducted according to the Declaration of Helsinki, as it involves human participants [15].

## Results

The program Swasthya Pahal aims to enhance the self-management of NCDs, including diabetes, hypertension, and obesity, in the rural and urban setting of Tamil Nadu, which will help in examining the individual’s perception of NCDs and also help in behavioral changes among the general population. The data collected will determine the predictors of the outcome variables of usefulness, acceptance, and effectiveness of Swasthya Pahal and broaden the applicability of the Swasthya Pahal program among individuals from rural and urban settings.

## Discussion

### Overview

NCDs claim the lives of 40 million people worldwide each year, accounting for 70% of all fatalities. Cardiovascular illnesses (17.7 million deaths), malignancies (8.8 million deaths), respiratory diseases (3.9 million deaths), and diabetes (1.6 million deaths) account for 81% of these deaths [16,17]. NCDs have become a serious public health problem in developing nations like India. NCD imposes financial consequences for India’s poorer households, as well as high out-of-pocket costs for the acute and long-term impacts of NCDs. This results in catastrophic health expenditure for households [18]. As a result, it is critical to screen individuals for NCDs and provide health education. Tailored ICT-supported NCD management programs such as Swasthya Pahal (Health for All) can make a difference by ascertaining an individual’s risk factor profile, as well as their perspective on NCDs and their health status. The critical need of the hour is the applicability of tailored NCD management interventions that can enhance knowledge and prognosis of the disease to the patient while also influencing policy decisions among low- and middle-income countries [19]. This digital platform intervention across a wider population group cannot only enhance the self-management or prevention of NCDs in individuals but its usefulness and acceptance may assist researchers and policymakers in health education and communication across the country.

The limitation of the study is that it is cross-sectional, needs to evaluate the long-term impact of the Swasthya Pahal program, and covers a large population group in the self-management of NCDs.

### Conclusions

Swasthya Pahal offers a personalized self-management plan that is supported by continuous monitoring, interactive health data, and collaborative decision-making. It focuses on an individual’s risk factor profile and helps us understand how they perceive NCDs and their health status. The research will help us understand the acceptability and usefulness of Swasthya Pahal among a large sample from urban and rural settings. It can be used to raise awareness and address the burden of NCDs. The program can be used for developing cost-effective interventions that can be used to educate populations from remote or unreached areas.

## Appendix

Multimedia Appendix 1 Study tools.

## Abbreviations

ICT: Information and Communication Technology
KAP: Knowledge, Attitudes, and Practices
NCD: noncommunicable diseases
SMAART: Sustainable, Multisector, Accessible, Affordable, Reimbursable, and Tailored framework

## Data Availability

The data sets generated during and/or analyzed during this study are available from the corresponding author on reasonable request.
