# Supplementary material for: Swasthya Pahal (Health for All) Using a Sustainable, Multisector, Accessible, Affordable, Reimbursable, and Tailored Informatics Framework in Rural and Urban Areas of Chennai, Tamil Nadu: Protocol for a Quantitative Study
Source: JMIR Res Protoc. 2025 Feb 6;14:e39950. doi: 10.2196/39950 (PMC11843048; doi:10.2196/39950)
Supplement: Multimedia Appendix 1 [file resprot_v14i1e39950_app1.docx]

## INFORMED CONSENT FORM

# **Title:** Usefulness, Acceptance, And Effectiveness of Swasthya Pahal Program Using SMAART Informatics Framework In Rural And Urban Areas Of Chennai, Tamil Nadu: Protocol For A Quantitative Study

**Objective**:

The objective of the study is to determine the usefulness, acceptance, and effectiveness of the Swasthya Pahal program in hospital and community settings in both rural and urban areas of Chennai, Tamil Nadu. The study will also help in generating risk factor profiles of individuals enrolled and enhancing their self-management of NCDs using a portable health information kiosk that employs the SMAART^TM^ model.

**Informed Consent**

I understand that I am being asked to participate in this study to facilitate the researchers to examine the usefulness, acceptance, and effectiveness of mobile technology-based program to enhance self-management of NCDs.

I understand that my participation in the study is voluntary and that it requires me to provide information on my socio-demographics, health behaviors, self-report assessment of their clinical history and objective assessments using weight, blood pressure and random blood sugar levels using series of physiological sensors. Additionally, questions for usability and acceptance will also be asked.

I agree to take part in the study, and I also authorize the distribution of my picture for educational and research purpose only. I understand that I am free to leave the study at any point of time and that the information provided by me will be used for research purposes and the results of the study will be included in journal publications and may be presented in the conferences but will not include any identifiers and personnel information will be kept confidential.

Signature of Field Staff: _____________________

Name of the Respondent with signature: ____________________

Signature/Thumb Impression: __________________

Audio consent provided: Yes/No

Mobile/Phone: _____________________

Address: _______________________________________________________________________________________________________________________________

Date and Time: ________________________

Annexure 1: Swasthya Pahal will collect socio-demographic information, clinical status information, and health behaviour information in a digital platform where researchers or individuals can access the information using a unique ID generated at the time of the study.

Step 1: Information about me

1. What is the location of your residence? Select one that applies

a. Urban

b. Slum

c. Village

d. Tribal

2. What is your Age in Years? Select one that applies

a. Years

b. I don’t know

3. What is your gender? Select one that applies

a. Male

b. Female

4. What is the highest level of education you had? Select one that applies

a. Less than high school

b. High School

c. Graduate

d. Post graduate

e. Professional

f. None

5. Do you smoke? Select one that applies

a. Presently

b. In the past

c. Never

If a or b Yes, how many cigarettes/cigars/bidis do you smoke on a weekly basis?

per week

6. Do you drink alcohol? Select one that applies

a. Presently

b. In the past

c. Never

6a.If a or b Yes, how many drinks of alcohol do you take on a weekly basis?

per week

7. Do you ever exercise?

a. Yes

b. Sometimes

c. No

7a. If a or b yes, how many times a do you exercise?

Times per week

Step 2: My Clinical Parameters

1. Have you ever been told by your doctor that you have diabetes/High blood sugar?

a. Yes

b. No

c. I don’t know

If b or c, Skip to Question 2

1a. If Yes, are you currently being treated for your high blood sugar?

a. Yes

b. No

c. I don’t know

If 1a. Yes, how do you manage your high blood sugar? Select all that apply

a. Medication

b. Insulin injection

c. Diet

d. Exercise

e. None of these options

2. Have you ever been told by your doctor that you have hypertension/High blood pressure?

a. Yes

b. No

c. I don’t know

If b or c, Skip to Question 3

2a. If Yes, are you currently being treated for your hypertension/High blood pressure?

a. Yes

b. No

c. I don’t know

2b. If 2a. Yes, how do you manage your high blood pressure? Select all that apply

a. Medication

b. Diet

c. Exercise

d. None of these options

3. Have you ever been told by your doctor that you have Hypercholesterolemia/High blood cholesterol?

a. Yes

b. No

c. I don’t know

If b or c, Skip to Question 4

3a. If Yes, are you currently being treated for your hypertension/High blood pressure?

a. Yes

b. No

c. I don’t know

3b. If 3a. Yes, how do you manage your high blood pressure? Select all that apply

a. Medication

b. Diet

c. Exercise

d. None of these options

4.Did anyone in your family have the following? Select all that apply

a. Diabetes

b. High blood pressure

c. High blood cholesterol

d. Heart disease

e. Other

f. None of them

Step 3: Measurement of Height

1.What is your height?

feet inches

Step 4: Measurement of Weight

2. What is your weight?

Kg

Step 5: BMI feedback

3.Your BMI IS: .

3a. Please select one option to tell if you are:

a. Underweight (<18.5)

b. Normal (18.5 – 24.9)

c. Overweight (25 – 29.9)

d. Obese (>=30)

e. I don’t know

Step 6: My blood pressure

4. Please enter your latest blood pressure reading

Systolic Diastolic Pulse

Step 6: Blood Pressure Feedback

4a. Your Blood Pressure is:

Please select one option to tell if your blood pressure is:

a. Normal (<120/80)

b. Pre-hypertension (>120/80)

c. Hypertension (>140/90)

d. I don’t know

Step 7: My Blood Sugar

5. Did you eat anything today?

a. Yes

b. No

5a. Please enter your latest blood sugar reading

Mg/dl

Step 7: Blood Sugar Feedback

6. Have you had your A1C Tested in the last 3 months?

a. Yes

b. No

c. I don’t know what A1C is

6a. If Q6 Yes, What was your A1C value?

a. mmol/mol

b. I don’t know

Step 7: Blood Sugar Feedback

Your Blood Sugar is: .

Please select one option to tell if your blood sugar is:

a. Normal(<140mg/dl)

b. Abnormal(>140mg/dl)

c. I don’t know

Step 8: My Blood Cholesterol level?

mg/dl

Your Blood Cholesterol is: .

Please select one option to tell if your blood cholesterol is:

a. Normal(<200mg/dl)

b. Abnormal(>=200mg/dl)

c. I don’t know

### Annexure 2: Tool for Acceptance: CSQ-8

Instructions for participants:

Please help us improve our service by answering some questions about the help that you have received.

We are interested in your honest opinions, whether they are positive or negative. Please answer all of the questions. We also welcome your comments and suggestions. Thank you very much. We appreciate your help.

1. How would you rate the quality of service you received?

- Excellent (4)
- Good (3)
- Fair (2)
- Poor (1)

2. Did you get the kind of service you wanted?

- No, definitely not (1)
- No, not really (2)
- Yes, generally (3)
- Yes, definitely (4)

3. To what extent has our service met your needs?

- Almost all of my needs have been met (4)
- Most of my needs have been met (3)
- Only a few of my needs have been met (2)
- None of my needs have been met (1)

4. If a friend were in need of similar help, would you recommend our service to him or her?

- No, definitely not (1)
- No, I don’t think so (2)
- Yes, I think so (3)
- Yes, definitely (4)

5. How satisfied are you with the amount of help you received?

- Quite dissatisfied (1)
- Indifferent or mildly dissatisfied (2)
- Mostly satisfied (3)
- Very satisfied (4)

6. Have the services you received helped you to deal more effectively with your problems?

- Yes, they helped a great deal (4)
- Yes, they helped somewhat (3)
- No, they really didn’t help (2)
- No, they seemed to make things worse (1)

7. In an overall, general sense, how satisfied are you with the service you received?

- Very satisfied (4)
- Mostly satisfied (3)
- Indifferent or mildly dissatisfied (2)
- Quite dissatisfied (1)

8. If you were to seek help again, would you come back to our service?

- No, definitely not (1)
- No, I don’t think so (2)
- Yes, I think so (3)
- Yes, definitely (4)

### Annexure 3: Tool for usability: System Usability Scale

| SYSTEM USABILITY SCALE | | Strongly disagree | Disagree | Neutral | Agree | Strongly agree |
| --- | --- | --- | --- | --- | --- | --- |
| 1 | I think I would like to use this program frequently |  |  |  |  |  |
| 2 | I found the program unnecessarily complex |  |  |  |  |  |
| 3 | I thought the program was easy to use |  |  |  |  |  |
| 4 | I think I would need the support of a technical person to be able to use this program |  |  |  |  |  |
| 5 | I found this program was well-integrated |  |  |  |  |  |
| 6 | I thought there was too much inconsistency in this program |  |  |  |  |  |
| 7 | I would imagine that most people that most people would learn to use this program quickly |  |  |  |  |  |
| 8 | I found the program very cumbersome/awkward to use |  |  |  |  |  |
| 9 | I felt confident using the program |  |  |  |  |  |
| 10 | I needed to learn a lot of things before I could go with this system |  |  |  |  |  |
